# *Enterocloster clostridioformis* protects against *Salmonella* pathogenesis and modulates epithelial and mucosal immune function

**DOI:** 10.1186/s40168-025-02050-9

**Published:** 2025-02-28

**Authors:** Benjamin S. Beresford-Jones, Satoshi Suyama, Simon Clare, Amelia Soderholm, Wangmingyu Xia, Puspendu Sardar, Junhee Lee, Katherine Harcourt, Trevor D. Lawley, Virginia A. Pedicord

**Affiliations:** 1 Jeffrey Cheah Biomedical Centre, Cambridge Institute of Therapeutic Immunology and Infectious Disease, Cambridge Biomedical Campus, Cambridge, UK; 2 Department of Medicine, University of Cambridge School of Clinical Medicine, Cambridge Biomedical Campus, Cambridge, UK; 3 Wellcome Sanger Institute, Wellcome Genome Campus, Hinxton, UK

**Keywords:** Acute salmonellosis, Microbiota, Pathogen resistance, Host–commensal interactions, *Enterocloster clostridioformis*, *Escherichia coli*, Mouse models, Screen, Resistin-like molecule β, T-regulatory cells

## Abstract

**Background:**

Promoting resistance to enteric pathogen infection is a core function of the gut microbiota; however, many of the specific host–commensal interactions that mediate this protection remain uncharacterised. To address this knowledge gap, we monocolonised germ-free mice with mouse-derived commensal microbes to screen for microbiota-induced resistance to *Salmonella* Typhimurium infection.

**Results:**

We identified *Enterocloster clostridioformis* as a protective species against *S.* Typhimurium infection. *E. clostridioformis* selectively upregulates resistin-like molecule β and cell cycle pathway expression at the level of caecal epithelial cells and increases T-regulatory cells in the underlying mucosal immune system, potentially contributing to reduced infection-induced pathology.

**Conclusions:**

We highlight novel mechanisms of host–microbe interactions that can mediate microbiota-induced resistance to acute salmonellosis. In the backdrop of increasing antibiotic resistance, this study identifies novel potential avenues for further research into protective host responses against enteric infections and could lead to new therapeutic approaches.

Video Abstract

**Supplementary Information:**

The online version contains supplementary material available at 10.1186/s40168-025-02050-9.

## Background

Non-typhoidal salmonellosis (NTS) is a major global health concern, causing 93 million enteric infections and over 150,000 deaths each year [1, 2]. Furthermore, NTS results in 4.07 million disability-adjusted life years, more than any other foodborne infection [3]. Antimicrobial resistance is an increasing concern globally, and drug-resistant salmonellae are now spreading in the human food supply chain increasing the financial burden of disease and worsening infection outcomes [4–6]. Effective treatment strategies and alternative approaches are therefore urgently required.

The gut microbiota has been implicated in providing colonisation resistance against *Salmonella* infection since Margaret Bohnhoff and colleagues demonstrated that streptomycin greatly increased susceptibility to infection in mice in 1954 [7]. Colonisation resistance has since been extensively studied, and many mechanisms have been identified by which commensal bacteria can inhibit colonisation of enteric pathogens. These include competition for metabolites including carbon sources, iron, oxygen, and electron acceptors [8–17], production of inhibitory substances [18, 19], and induction of host immune responses that control pathogen titres [20–22].

However, the microbiota also contributes to alternative mechanisms beyond colonisation resistance that protect hosts from enteric infections. Commensal microbes can directly modulate pathogen virulence [10, 23–26] or induce host responses that increase resistance to disease without directly affecting the infectious titres in the intestinal lumen. While some examples of these microbiota-induced host responses have been described — including modulation of epithelial signalling and instigation of mucosal immune responses to induce resistance to infection and tolerance to immunopathology [27–30] — it is likely that many more mechanisms of commensal-mediated enteric pathogen resistance remain unknown [27].

In this study, we identified a novel role for the commensal bacterium *Enterocloster clostridioformis* in the induction of protective responses in a mouse model of acute *Salmonella* enterocolitis. We demonstrate that *E. clostridioformis* induces a unique transcriptional response in caecal epithelial cells, upregulating resistin-like molecule β and pathways associated with cell cycle, and elicits an increase in local regulatory T-cell populations. These host effects correlate with decreased tissue pathology and significantly prolonged survival in germ-free mice. Collectively, these findings suggest that *E. clostridioformis* may reduce pathology following *Salmonella* infection by inducing protective caecal epithelial responses and increasing regulatory T cells in the mucosal epithelium, although these mechanisms have not been causally established in the present study.

## Results

### An in vivo screen reveals* E. clostridioformis-*mediated protection against *Salmonella* infection

Mice infected with *Salmonella enterica* serovar Typhimurium (*S.*Tm) do not develop robust gut inflammation akin to acute NTS but instead develop a systemic, typhoid fever-like disease [31, 32]. In contrast, antibiotic pretreatment prior to *S.*Tm infection triggers an inflammatory diarrhoeal disease which mimics acute NTS in humans and can be used as a mouse model for *Salmonella* enterocolitis [7, 32–34]. We therefore initially attempted to optimise a specific pathogen-free (SPF) mouse model for *S.*Tm infection through administration of different broad-spectrum antibiotic cocktails via intragastric gavage or in drinking water.

We observed that different antibiotic treatment regimens resulted in inconsistent levels of susceptibility to infection (Supplementary Fig. 1A). Survival significantly correlated with the degree to which the microbiota recovered following antibiotic administration (Supplementary Fig. 1B), and there was significant variation between cohorts even after using the same route and regimen of antibiotics, limiting reproducibility of these experiments (Supplementary Fig. 1C). In contrast, germ-free (GF) mice were invariably sensitive to *S.*Tm infection, with pathogen titres of < 100 CFU/mouse resulting in rapid weight loss and complete mortality within 2 days of infection. Importantly, this finding was reproducible across experiments.

**Fig. 1:**
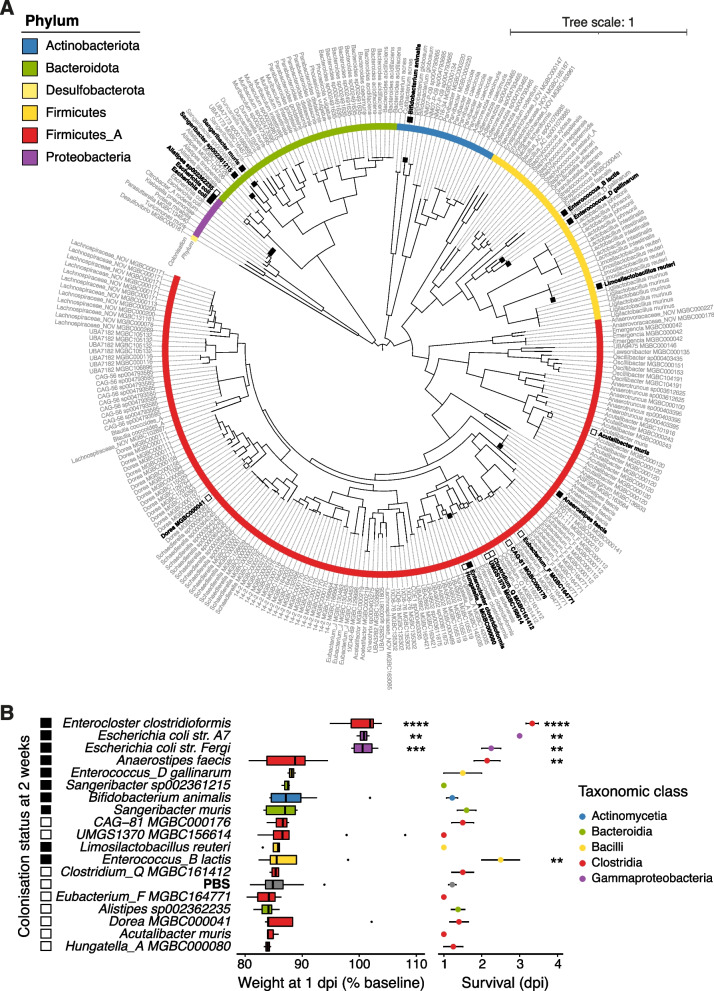
Screened isolates of the Mouse Culture Collection. **A** Maximum-likelihood phylogenetic tree of the 276 isolates of the MCC in addition to the genome of *Enterococcus_B lactis* str. Com15 (formerly *E. faecium*). Distances represent BLOSUM45 matrix analysis of amino acid alignments of 120 bacterial core genes using the JTT + CAT model. Screened isolates are indicated in black boldface type. Inner colour bar indicates the taxonomic phylum of isolates. Filled black boxes illustrate isolates that successfully colonised mice after 14 days monocolonisation, while filled white boxes indicate species that were administered to mice but could not be recovered from faeces after 14 days. **B** Monocolonisation with commensal isolates prior to *Salmonella* Typhimurium infection. From left to right: species name, filled boxes demarcate isolates that were recovered from the faeces 14 days after inoculation, mouse weights at 1 dpi as a percentage of starting weights (0 dpi), mouse survival post-infection. Data shown are pooled from six screening experiments (*n* = 2–18). Wilcoxon tests comparing commensal isolate-monocolonised mice to PBS controls with Benjamini–Hochberg correction

We therefore performed a screen of mouse gut-derived commensal microbes in GF mice to identify causative microbe–host interactions that are protective against *S.*Tm infection. GF mice aged 6 to 8 weeks old were monocolonised via intragastric administration with a single, high-titre inoculum of a commensal bacterial species isolate from our Mouse Culture Collection [35]. Following 14 days of monocolonisation, mice were infected with *S.*Tm 14028, and weight loss at 1-day post-infection and time to reach 80% of baseline weight were used as proxies for infection severity. A total of 18 commensal bacterial isolates from the Mouse Culture Collection were successfully screened for their impact on *S.*Tm infection (Table 1), including representatives from five taxonomic phyla (Fig. 1A). Importantly, no monocolonised mice suffered any mortality or exhibited any manifestations of disease prior to infection.

**Table 1:** Isolates of the MCC used to screen for resistance against *S.* Typhimurium infection

Species	Strain	Phylum	Class	Order	Family
*Acutalibacter muris*	B37	Firmicutes_A	Clostridia	Oscillospirales	*Acutalibacteraceae*
*Alistipes* sp002362235	A61	Bacteroidota	Bacteroidia	Bacteroidales	*Rikenellaceae*
*Anaerostipes faecis*	A14	Firmicutes_A	Clostridia	Lachnospirales	*Lachnospiraceae*
*Bacteroides* sp002491635	A10	Bacteroidota	Bacteroidia	Bacteroidales	*Bacteroidaceae*
*Bifidobacterium animalis*	A82	Actinobacteriota	Actinomycetia	Actinomycetales	*Bifidobacteriaceae*
*Sangeribacter* sp002361215	A17	Bacteroidota	Bacteroidia	Bacteroidales	*Muribaculaceae*
*Sangeribacter muris*	A43	Bacteroidota	Bacteroidia	Bacteroidales	*Muribaculaceae*
CAG-81 MGBC000176	C57	Firmicutes_A	Clostridia	Lachnospirales	*Lachnospiraceae*
*Clostridium*_Q MGBC161412	C48	Firmicutes_A	Clostridia	Lachnospirales	*Lachnospiraceae*
*Dorea* MGBC000041	A26	Firmicutes_A	Clostridia	Lachnospirales	*Lachnospiraceae*
*Duncaniella muricolitica*	A60	Bacteroidota	Bacteroidia	Bacteroidales	*Muribaculaceae*
*Emergencia* MGBC000042	A28	Firmicutes_A	Clostridia	Peptostreptococcales	*Anaerovoracaceae*
*Enterocloster clostridioformis*	A92	Firmicutes_A	Clostridia	Lachnospirales	*Lachnospiraceae*
*Enterococcus_D gallinarum*	A2	Firmicutes	Bacilli	Lactobacillales	*Enterococcaceae*
*Escherichia coli*	A7	Proteobacteria	Gamma-proteobacteria	Enterobacterales	*Enterobacteriaceae*
*Escherichia coli*	Fergi	Proteobacteria	Gamma-proteobacteria	Enterobacterales	*Enterobacteriaceae*
*Eubacterium*_F MGBC164771	A22	Firmicutes_A	Clostridia	Lachnospirales	*Lachnospiraceae*
*Hungatella*_A MGBC000080	A68	Firmicutes_A	Clostridia	Lachnospirales	*Lachnospiraceae*
*Ligilactobacillus murinus*	A9	Firmicutes	Bacilli	Lactobacillales	*Lactobacillaceae*
*Limosilactobacillus reuteri*	A12	Firmicutes	Bacilli	Lactobacillales	*Lactobacillaceae*
*Parabacteroides goldsteinii*	A19	Bacteroidota	Bacteroidia	Bacteroidales	*Tannerellaceae*
*Schaedlerella* sp000364245	B8	Firmicutes_A	Clostridia	Lachnospirales	*Lachnospiraceae*
UMGS1370 MGBC156614	C49	Firmicutes_A	Clostridia	Lachnospirales	*Lachnospiraceae*

Although all isolates were successfully cultured from the inoculum after oral gavage, only 10 isolates stably colonised and were viably recovered from the faeces after 14 days of monocolonisation. Most of the unrecovered species belonged to the class Clostridia (*n* = 7; phylum Firmicutes_A), while one, *Alistipes* sp002362235, was a member of the class Bacteroidia. Successful colonisation with any isolate tended to marginally reduce weight loss at 1-day post-infection, as PBS controls lost more weight on average than any successfully monocolonised mice. However, *E. clostridioformis* and two *Escherichia coli* strains, A7 and Fergi, significantly reduced weight loss at 1-day post-infection and significantly increased survival compared to PBS controls (Fig. 1B). Two additional isolates, *Anaerostipes faecis* and *Enterococcus_B lactis*, also significantly improved mouse survival compared to PBS controls but did not reduce weight loss at 1-day post-infection.

### *E. clostridioformis* elicits tissue protection without preventing *Salmonella* colonisation

**Fig. 2:**
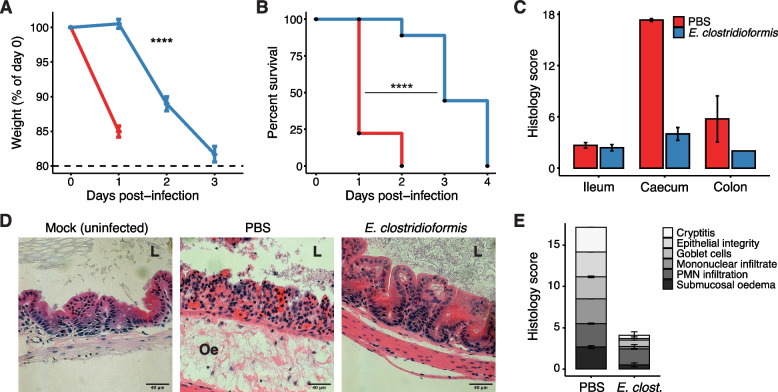
*E. clostridioformis* reduces severity of *S.* Typhimurium infection in germ-free mice. **A** Weight loss and **B** survival following infection with 10^2^ CFU/mouse *S*.Tm. Weight loss data represent the mean ± SEM; means are not shown for timepoints after any mouse within the group was euthanised. Survival represents the day at which mice reached 80% of pre-infection weight. Kruskal–Wallis and log-rank tests were used for statistical analyses in **A** and **B**, respectively. Data shown are pooled from six experiments (*n* = 18). **C** Cumulative pathology scores for H&E-stained sections of the ileum, caecum, and colon of GF or *E. clostridioformis*-monocolonised mice at 1 dpi. Representative data shown are from one of two experimental repeats, mean ± SEM of cumulative pathology scores from individual mice (*n* = 2–4). **D** Representative sections of the caecum at 1 dpi (PBS, *E. clostridioformis*) or from noninfected GF mice (Mock). L, caecal lumen; Oe, submucosal oedema. Scale bars indicate 40 μm. **E** Breakdown of the caecum pathology scores at 1 dpi according to the six contributing subcomponents, mean ± SEM. Representative data shown are from one of two experimental repeats (*n* = 2–4)

The *Enterobacteriaceae* family as well as individual strains of *E. coli* such as Nissle 1917 have previously been associated with protection against *S.*Tm infection in mice through colonisation resistance. Indeed, *E. coli* has become a model organism for understanding colonisation resistance in *S.*Tm infection [36]. In contrast, *E. clostridioformis* monocolonisation has not been previously implicated in protection against *S.*Tm infection, and we therefore sought to better understand this association. Although all mice did eventually proceed to mortality, monocolonisation with *E. clostridioformis* significantly increased resistance to *S.*Tm infection, delaying and reducing the rate of weight loss (Fig. 2A) and increasing survival (Fig. 2B). Mortality at 1-day post-infection was reduced from 76% in germ-free mice to 0% following *E. clostridioformis* treatment (Fig. 2B).

*S.*Tm produces virulence factors which it both secretes and directly injects into host intestinal epithelial cells (IECs) via its SPI-1-encoded type III secretion system to mediate invasion, resulting in an acute inflammatory response. Accordingly, at 1-day post-infection, GF mice exhibited extensive pathology in the caecum (Fig. 2C), with extreme submucosal oedema, complete loss of epithelial integrity, and goblet cell depletion (Fig. 2D, E). In comparison, *E. clostridioformis*-monocolonised mice displayed markedly reduced caecal inflammation and pathology at 1-day post-infection (Fig. 2C, D, E). Although some pathological changes were seen in the colons of GF mice following *S.*Tm infection, most notably submucosal oedema, these were not as profound as in the caecum, and no mice from either cohort exhibited extensive pathology in the terminal ileum (Figs. 2C, D, E). This aligns with previous reports that invasion and pathology at early time points after *S.*Tm infection predominantly occur in the caecum and colon [33].

**Fig. 3:**
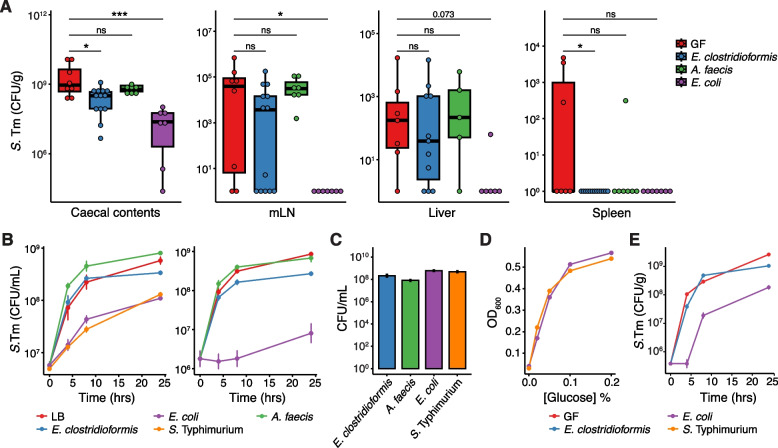
*E. clostridioformis* protects germ-free mice against *S.* Typhimurium infection independently of colonisation resistance. **A** *S*.Tm titres (CFU/g tissue) at 1 dpi in caecum luminal contents, mLN, liver, and spleen (left to right). Each data point represents a single mouse; Wilcoxon tests were used to assess statistical significance, and the Benjamini–Hochberg procedure were used to correct for multiple comparisons. Data shown are pooled from three experimental repeats (*n* = 5–13). **B** Titres of *S*.Tm following inoculation of filter-sterilised spent media (left) or stationary phase media (right) from commensal isolates. Data are pooled from 2 to 3 experimental repeats (*n* = 3–9). **C** Commensal and *S*.Tm titres at 24 h in LB broth under anaerobic conditions Pooled data are shown from two experiments (*n* = 6). **D** Growth of *S*.Tm in sterile-filtered spent media following addition of glucose. Data shown from one experiment (*n* = 3). **E** Growth of *S*.Tm following inoculation of caecal contents from GF or commensal-monocolonised mice. Representative data shown are from one of three experiments (*n* = 2–5)

Following on from the histological phenotype, we next considered the extent of intestinal infection and *S.*Tm dissemination to peripheral organs in mice colonised with *E. clostridioformis*, *A. faecis*, or *E. coli* (strain A7). *A. faecis* was included as a negative control as it is the closest taxonomically related organism to *E. clostridioformis* successfully included in our screen and did not reduce weight loss following acute *S.*Tm infection. Compared to GF mice, *S.*Tm titres in the caecal lumen at 1-day post-infection were lower in both *E. coli*- and *E. clostridioformis*-monocolonised mice than in GF controls (Fig. 3A), although titres were significantly lower in the *E. coli* group compared to *E. clostridioformis* (38.5-fold reduction vs 2.9-fold reduction, *p* = 0.008). This reduced *S.*Tm titre in the presence of *E. clostridioformis* is in keeping with previous research demonstrating that *E. clostridioformis* and *S.*Tm completely overlap in their ability to metabolise C5 and C6 sugars [37], suggesting that *E. clostridioformis* may compete with *S.*Tm in this niche. However, *S.*Tm titres in *E. clostridioformis*-monocolonised mice remained high enough to mediate tissue invasion, as there was no difference in titres of *S.*Tm in mesenteric lymph nodes (mLN) or liver compared to GF mice (Fig. 3A). Splenic titres were undetectable in both *E. coli*- and *E. clostridioformis*-monocolonised mice as well as most *A. faecis*-colonised control mice (Fig. 3A).

Colonisation resistance is the best described mechanism for commensal-mediated protection against *S.*Tm, and *E. coli* is well-known for its capacity to protect against *S.*Tm through mechanisms of colonisation resistance [17, 37]. Therefore, we next assessed whether *E. clostridioformis* also mediates resistance to infection by colonisation resistance. *S.*Tm growth was significantly attenuated when grown in both stationary phase broth culture and sterile-filtered spent media from *E. coli*, but not in media from *E. clostridioformis* or *A. faecis* (Fig. 3B). The commensal isolates used for these assays grew to a similar extent and exhibited minimal variation in culture titres (Fig. 3C). The addition of glucose to spent media from *E. coli* and *S.*Tm supplemented *S.*Tm growth in a concentration-dependent manner (Fig. 3D), indicating that reduced stationary phase titres were mediated by carbon source depletion rather than media toxification.

We next performed ex vivo faecal spiking assays to confirm the absence of a colonisation resistance phenotype in a more physiologically relevant setting. For this assay, the caecal contents of mice monocolonised with either *E. coli* or *E. clostridioformis* were inoculated with *S.*Tm and incubated anaerobically at 37 °C. While *E. coli* monocolonisation significantly delayed and reduced *S.*Tm growth, *S.*Tm growth in *E. clostridioformis*-monocolonised caecal contents was equivalent to growth in caecal contents from GF mice (Fig. 3E). Taken together, these findings strongly suggest that the protective phenotype of *E. clostridioformis* against *S.*Tm is not primarily mediated by colonisation resistance.

Finally, we considered the impact of *E. clostridioformis* on *S.*Tm virulence. Although systemic dissemination of *Salmonella* has also been shown to occur independently of SPI-1 [38], there was no difference in *S.*Tm virulence gene expression between bacteria grown in GF or *E. clostridioformis*-monocolonised caecal contents (Supplementary Fig. 2). However, *E. clostridioformis* monocolonisation was associated with less contact between *S.*Tm and the caecal epithelium in vivo (Supplementary Fig. 3A, B). While there was also a trend towards decreased *S.*Tm invasion of epithelial cells following pretreatment with *E. clostridioformis-* conditioned media in vitro, this was not statistically significant and not specific to *E. clostridioformis* as the same decreasing trend was observed with *E. coli* (Supplementary Fig. 3C). Collectively, the data suggest that *E. clostridioformis* does not directly impact *S.*Tm virulence, indicating that additional host mechanisms are required to mediate the protective phenotype.

### Bacterial colonisation induces differential host transcriptional responses in the caecal epithelium

**Fig. 4:**
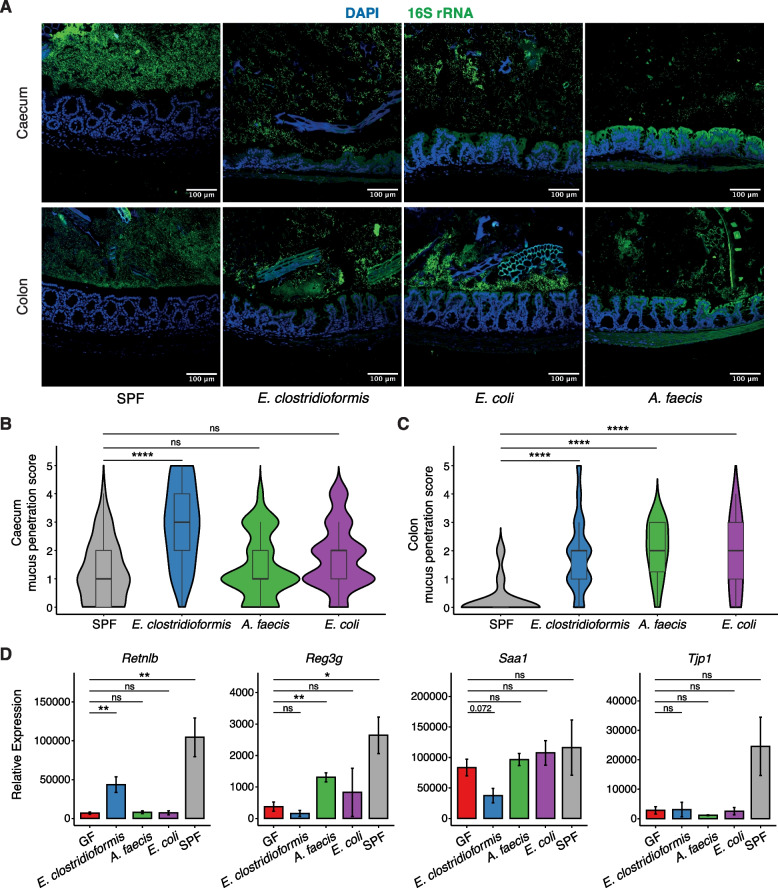
*E. clostridioformis* is closely associated with the caecal epithelium and induces expression of RELMβ. **A** Representative micrographs showing caecal (top row) and colonic (bottom row) epithelia from SPF mice and mice monocolonised with *E. clostridioformis*, *E. coli*, and *A. faecis*. Epithelial nuclei are stained with DAPI (blue), and bacteria are stained with a universal 16S rRNA gene FISH probe conjugated to FITC (green). Scale bars indicate 100 μm. **B**, **C** Epithelial proximity scores of the caecum (**B**) and colon (**C**). Scoring was performed by an independent, blinded investigator. Data are pooled from five experiments (*n* = 4–7) Wilcoxon tests with Benjamini–Hochberg correction. **D** Relative expression by qPCR of host defence genes in caecal epithelial cells isolated from GF, SPF, or *E. clostridioformis*-, *E. coli*-, or *A. faecis*-monocolonised mice. Data shown are pooled from six experiments (*n* = 4–14). Student’s *t*-tests with Benjamini–Hochberg correction

Given the lack of colonisation resistance observed, we examined the association of the commensal microbes with the host epithelium. We performed fluorescence in situ hybridisation (FISH) with a universal 16S rRNA gene probe to visualise commensal microbes in the context of the intestinal epithelium in mice monocolonised with *E. clostridioformis*, *E. coli*, or *A. faecis* (Fig. 4A). Use of a modified epithelial proximity score indicated that *E. clostridioformis* was significantly more closely associated with the caecal epithelium than the multispecific microbiota of SPF mice (Fig. 4B). This was not the case for *E. coli* and *A. faecis* which were not significantly different. This trend did not continue into the colon, as epithelial proximity was greater by all species compared to SPF mice (Fig. 4C).

Based on this close association with the epithelium, we investigated epithelial expression of genes associated with epithelial barrier function and antimicrobial responses. We performed RT-qPCR on IECs from caeca of GF and SPF mice, as well as mice monocolonised with *E. clostridioformis*, *E. coli*, or *A. faecis*. Monocolonisation with *E. clostridioformis* induced a sixfold increase in caecal IEC expression of the antimicrobial peptide resistin-like molecule β (RELMβ, gene: *Retnlb*; Fig. 4D). This response was specific to *E. clostridioformis*, as *Retnlb* expression was not increased following monocolonisation with *E. coli* or *A. faecis,* and did not reflect a general response to bacterial colonisation (Fig. 4D). *E. clostridioformis* monocolonisation was not associated with increased expression of other barrier function genes we examined, including *Reg3g*, *Tjp1* (mZO-1), or *Muc2* (Fig. 4D, Supplementary Fig. 4A). Despite being closely associated with the caecal epithelium, expression of *Saa1* was reduced following *E. clostridioformis* monocolonisation compared to GF mice (Fig. 4D).

**Fig. 5:**
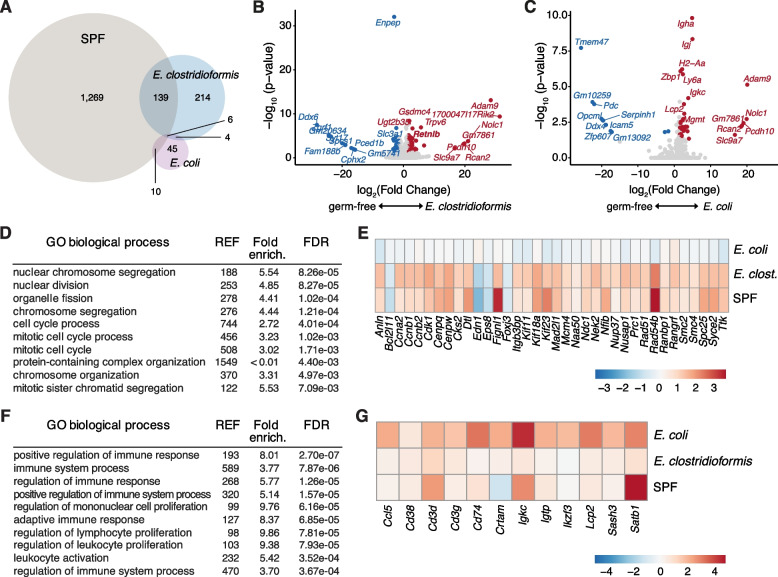
*E. clostridioformis* and *E. coli* induce specific transcriptional signatures in caecal epithelial cells. Bulk RNA sequencing of caecal epithelial cells isolated from SPF mice and mice monocolonised with either *E. clostridioformis* or *E. coli* compared to GF controls (*n* = 6 from three experiments). **A** Venn diagram of significantly differentially expressed genes. **B**, **C** Volcano plots of genes from *E. clostridioformis*- and *E. coli*-monocolonised mice compared to GF controls. Genes highlighted in red or blue are significantly differentially expressed (*FDR* ≤ 0.05) with a log_2_ fold change either ≥ 1.5 (red) or ≤ − 1.5 (blue). **D** Top 10 most significantly enriched GO terms following *E. clostridioformis* colonisation. **E** Heatmap showing genes from the cell cycle gene set that are differentially expressed following *E. clostridioformis* monocolonisation. **F** Top 10 most significantly enriched GO terms following *E. coli* colonisation. **G** Heatmap showing genes from the leukocyte activation gene set that are differentially expressed following *E. coli* monocolonisation

To gain a broader understanding of *E. clostridioformis*-induced changes in host epithelial function, we performed RNA sequencing-based transcriptomic analyses of caecal IECs from *E. clostridioformis* and *E. coli* monocolonised mice compared to GF and SPF controls. SPF mice exhibited 1424 significantly differentially expressed genes compared to GF mice, while *E. clostridioformis* monocolonisation resulted in differential expression of 363 genes compared to only 65 with *E. coli* (Fig. 5A). The largest portion of SPF genes were shared with *E. clostridioformis* (*n* = 139), with only 10 genes shared with *E. coli* and 6 genes shared by mice from all 3 groups (Fig. 5A). These data suggest that *E. clostridioformis* monocolonisation achieved partial normalisation of the IEC transcriptional landscape seen with a replete SPF microbiota, substantially more than that seen with *E. coli* monocolonisation. *Retnlb* remained significantly differentially expressed in the transcriptomic data for *E. clostridioformis* (Fig. 5B), while *E. coli* demonstrated significant upregulation of multiple immune-related genes (Fig. 5C).

To identify transcriptional pathways modulated by *E. clostridioformis* colonisation, we next performed GO term enrichment analyses. *E. clostridioformis* demonstrated a specific enrichment for GO terms linked to cell cycle processes (Fig. 5D, E; Supplementary Table 1), although tissue architecture remained similar to GF mice (Supplementary Fig. 4B, C). In contrast, *E. coli* upregulated multiple immune pathways, including adaptive immune response, regulation of leukocyte and lymphocyte proliferation, and leukocyte activation (Fig. 5F, G; Supplementary Table 1). These findings, taken together with the respective phenotypes for the commensal bacteria, suggest that *E. coli* directly competes with *S.*Tm to mediate colonisation resistance but also induces upregulation of immune pathways at the level of the host epithelium. In contrast, *E. clostridioformis* does not induce a pro-inflammatory transcriptional response in the epithelium but instead upregulates genes associated with cell cycle, potentially associated with restoration of the epithelium.

### Anti-inflammatory T-cell ratios are increased by *E. clostridioformis* colonisation

**Fig. 6:**
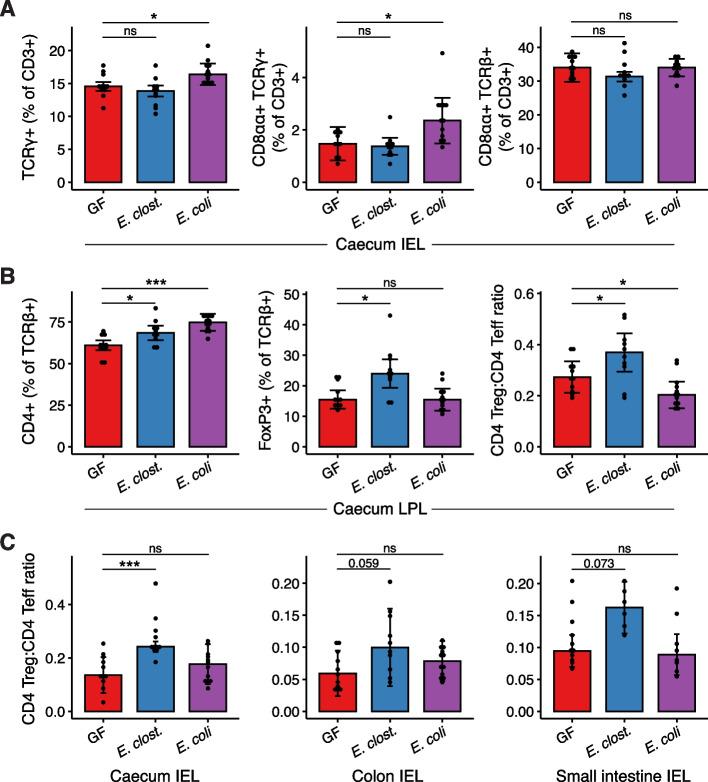
*E. clostridioformis* increases the proportion of Tregs in the caecal epithelium, while *E. coli* increases γδ-T cells. Lymphocyte populations were quantified for different intestinal compartments using flow cytometry. Pooled data are shown from three experiments (*n* = 6–12). **A** Lymphocytes from the caecal intraepithelial compartment. **B** Lymphocytes from the caecal lamina propria. **C** Ratios of Foxp3 + CD4 + T-regulatory cells to Foxp3 − CD4 + T-effector cells from the caecal, colonic, and small intestinal intraepithelial compartments. Each data point represents a single mouse. Wilcoxon tests with Benjamini–Hochberg correction. Bars and error bars represent median ± median absolute deviation, respectively

In light of the upregulation of immune pathways by *E. coli*, we next sought to characterise the impact of monocolonisation on the intestinal immune system by flow cytometry. In the caecal intraepithelial (IEL) compartment, *E. coli* monocolonisation resulted in a significant increase in γδ-T cells, predominately represented by an increase in CD8αα + γδ-T cells (Fig. 6A). There were no changes in the abundance of these cell types following *E. clostridioformis* colonisation. CD8αα + αβ-T-cell abundance remained unchanged by both *E. clostridioformis* and *E. coli* colonisation (Fig. 6A).

In the caecum lamina propria, colonisation with either isolate resulted in a significant increase in CD4 + T cells (Fig. 6B). However, *E. clostridioformis* induced a selective increase in Foxp3 + CD4 + regulatory T cells (Tregs), resulting in an increased Treg:T-effector cell ratio (Fig. 6C). In contrast, *E. coli* colonisation did not impact Treg abundance, resulting in a significantly reduced Treg:T effector cell ratio (Fig. 6C). *E. clostridioformis* colonisation also increased the relative abundance of Tregs in the caecum IEL compartment and trended towards increased ratios in the colon and small intestine IEL compartments (Fig. 6C). Of note, B-cell, Treg, and effector T-cell populations in the colon lamina propria and mesenteric lymph nodes were not significantly affected by *E. clostridioformis* colonisation (Supplementary Fig. 5), indicating a more pronounced effect on the caecal immune repertoire. These data suggest that increased tolerogenic mucosal immune tone may be one mechanism by which *E. clostridioformis* reduces the pathological effects of *S.*Tm infection, although a direct causal role for Tregs in the protective phenotype remains to be demonstrated.

## Discussion

In this study, we utilised gut bacterial isolates from mice to screen for microbial species that confer resistance to *Salmonella* Typhimurium infection in a germ-free mouse model. Our findings reveal *E. clostridioformis* as a protective species against acute salmonellosis. Mice monocolonised with *E. clostridioformis* displayed reduced weight loss, prolonged survival, and decreased caecal and colonic pathology. This phenotype was associated with increased expression of the host defence peptide resistin-like molecule β (*Retnlb*/RELMβ) in caecal epithelial cells and increased abundance of CD4 + T-regulatory cells (Tregs) in the intraepithelial and lamina propria compartments. Altogether, these results highlight restoration of proliferation, antimicrobial peptide production, and possible induction of regulatory mucosal immune responses as a potential novel multifaceted mechanism of microbiota-induced host protection against infection-associated inflammation and tissue damage.

Several of our results indicate that the protective phenotype induced by *E. clostridioformis* is unlikely to be mediated by colonisation resistance. Firstly, the reduction in infection severity by *E. clostridioformis* is equivalent if not slightly greater to that of *E. coli*, despite *E. coli* exhibiting a much stronger colonisation resistance phenotype against *S.*Tm both in vivo and in vitro. Secondly, while caecum luminal titres are slightly reduced by *E. clostridioformis*, the *S.*Tm titres in the mLN and liver were not significantly different between germ-free and *E. clostridioformis* monocolonised mice, indicating that titres in the caecal lumen are still sufficient for tissue invasion and effective infection. In contrast, *E. coli* colonisation significantly reduced these titres. Instead of colonisation resistance, the upregulation of cell cycle pathways and increased abundance of Tregs in the caecum suggest that *E. clostridioformis* may increase tolerance to acute *S.*Tm infection through restoration of the epithelium and reduction of immune-mediated damage to host tissues, although further studies are required to establish a causal link.

Of note, our screen of commensal microbes used weight loss as a measure to detect protection against acute *Salmonella* pathology. However, the caeca of GF mice are grossly enlarged and can contribute multiple grams to their overall weight. The hyperacute weight loss seen during our screen is therefore more representative of *Salmonella*-induced caecal inflammation rather than systemic sickness. This is supported by the extensive submucosal oedema and high histology scores seen in GF mice as early as 1-day post-infection, as well as the similarities in systemic bacterial translocation between GF and *E. clostridioformis*-monocolonised mice. Our screen therefore preferentially selected for gut bacterial strains that protect from caecal inflammation rather than extend survival.

The increased expression of resistin-like molecule β (Retnlb/RELMβ) associated with *E. clostridioformis* colonisation may provide insights into the underlying molecular mechanisms of the protective phenotype. RELMβ is a host defence protein with antimicrobial activity against a range of both gram-negative and gram-positive bacteria [39, 40] as well as an important role in maintaining colonic epithelial barrier function [39, 41]. While it has not been previously studied in the context of *S.*Tm, RELMβ has been shown to have an important role in CD4 + T-cell recruitment and regulation of T-cell-dependent IEC proliferation in response to *Citrobacter rodentium* infection [42], and its expression in tumours is also linked to IEC proliferation [43]. As such, its upregulation in response to *E. clostridioformis* might provide a mechanistic link to the upregulation of cell cycle pathways seen in our bulk RNA sequencing analyses. More recently, RELMβ has been proposed as a marker of deep crypt secretory (DCS) cells in the mouse colon [44]. Interestingly, additional markers of DCS cells including Reg4, Spink4, and Agr2 [45, 46] were also significantly upregulated by *E. clostridioformis*, suggesting that monocolonisation with this bacterium may drive expansion and upregulation of the DSC cell niche. DSC cells have an important albeit incompletely elucidated role in maintaining colonic health [47]. These studies provide a theoretical basis for a pivotal role for RELMβ in the *E. clostridioformis*-induced host phenotype, although causality remains to be experimentally validated.

The importance of Tregs in maintaining a tolerogenic environment in the gut is well established. Tregs are known to be important in directing early adaptive immune responses to *S.*Tm infection [48] and are also reduced in GF mice [49–51]. Butyrate synthesis has been shown to constitute one mechanism by which commensal microbes can increase lamina propria Treg populations [52]; however, *E. clostridioformis* does not encode either terminal pathway required for producing butyrate, implying that it increases Tregs through a different mechanism. As our data indicate that *E. clostridioformis* monocolonisation increases the caecal Treg proportion of total CD4 + T cells to 37% — similar to published values for colonic lamina propria Tregs in SPF mice [50, 53] — further study of this microbe–host interaction beyond to its role in *S.*Tm resistance may be warranted.

A multitude of studies have described mechanisms for *E. coli* colonisation resistance against *S.*Tm, but few have commented on the role of the concomitant host response. As such, it remains unknown to what extent the host response to *E. coli* contributes to its protective phenotype. We have demonstrated that *E. coli* monocolonisation induces a pro-immune transcriptional signature and changes in mucosal immune cell subsets including γδ-T cells that are known to orchestrate protective epithelial responses in infection [54]. Although the specific contributions of these findings to *E. coli*-mediated pathogen resistance have not been addressed in this study, these findings underscore the complexity of host–microbiota interactions in pathogen defence and may form a foundation for future studies.

In our screen of multiple taxonomically-related and taxonomically-diverse commensal species, only *E. clostridioformis* was found to induce the potentially protective host responses we observed. However, we hypothesise that these phenotypes represent conserved responses to the gut microbiota and therefore will be shared by multiple commensals and across multiple host species. As such, the causative mechanisms that underpin these responses, including the relevant bacterial metabolites and host signalling pathways, warrant further investigation.

## Conclusions

In conclusion, our study identifies *E. clostridioformis* as a novel protective species against *S.*Tm infection and highlights new pathways and mechanisms that could contribute to host pathogen defence. Non-typhoidal *Salmonella* (NTS) represents an important global health concern, and rising antibiotic resistance necessitates the investigation of alternative approaches to disease prevention and treatment. Future research should focus on elucidating the mechanisms underlying this phenotype, including the specific microbial signals and intermediary effectors involved, potentially paving the way for novel approaches to prevent and treat NTS infection and other related pathologies.

## Methods

### Mice

Mice were maintained under either specific pathogen-free (SPF) or germ-free (GF) conditions at the Home Office-approved Wellcome Sanger Institute mouse facility, and all procedures were carried out in accordance with the United Kingdom Animals (Scientific Procedures) Act of 1986.

SPF mice were bred and maintained under barrier conditions in individually ventilated cages, which were then transferred from the barrier to a separate containment room prior to experiments to facilitate researcher access. Male and female mice were housed separately, and only female C57BL/6NTac mice aged 6 to 8 weeks were used for SPF infection experiments. Non-acidified autoclaved water and irradiated chow (Safe R03-10 complete breeding diet; crude protein: 21.4%, crude fat: 5.1%, crude ash: 5.4%, crude fibre: 4.0%) were provided for ad libitum consumption. All cages were provided with bedding, nesting material, and enrichment. Mice were exposed to a 12-h light–dark cycle. Cage cleaning was kept to a maximum of once per week, and clean/dry nesting material was transferred to new cages during cage change to maintain familiar odours. Cage cleaning was maintained during experiments, although cages were additionally changed following the initiation of antibiotic treatment to prevent coprophagy of microbially replete faecal pellets.

C57BL/6 J GF mice were bred and housed in sterile isolators prior to microbial colonisation or infection experiments. These mice were maintained in positive-pressure isolators and screened every week for contamination using faecal culture, microscopy, and 16S rRNA gene PCR. Consumables were autoclaved at 121 °C before introduction into the isolators. For experiments, cages were removed from isolators and opened in a vapourised hydrogen peroxide-sterilised class II safety cabinet. Mice were only handled under aseptic conditions. Mice were then maintained in sterilised bioexclusion cages (ISOcage P, Tecniplast) under positive pressure to prevent external contamination. Mice of both sexes aged between 6 and 9 weeks were used in GF infection studies. GF mouse experiments were performed in a containment level 3 facility.

### Bacterial strains

Selected isolates of the MCC were administered to GF mice to screen for resistance to *S.*Tm infection. The details of these isolates are included in Table 1. Non-MCC isolates were also included in this screen, namely *Enterococcus_B lactis* (formerly *Enterococcus faecium*) strain Com15 and *Clostridium_A leptum*. *S.*Tm strain 14028 (strain designation: CDC 6516–60) was purchased from the American Type Culture Collection specifically for use in the screening protocol. All isolates were stored at − 80 °C in 25% glycerol for medium-term storage. Where *E. coli* is used in this paper without a strain classifier, *E. coli* A7 was used. Bacterial strains used in this study will be made available upon request.

### Administration of antibiotics via oral gavage

A broad-spectrum antibiotic cocktail of ampicillin (20 mg/mL), vancomycin (10 mg/mL), metronidazole (10 mg/mL), neomycin (20 mg/mL), and ciprofloxacin (3 mg/mL) was prepared using autoclaved drinking water and by stirring and heating to a maximum of 42 °C. Antibiotic solutions were sterile filtered using a 0.22-µm filter prior to administration to mice.

To perform oral gavage, mice were anaesthetised with isoflurane before receiving 200 µL of antibiotic solution intragastrically via a metal ball-tipped feeding needle. Oral gavage was performed each morning daily for 7 days using fresh preparations of the antibiotic cocktail.

### Administration of antibiotics in the drinking water

Ampicillin (1 g/L), vancomycin (0.5 g/L), metronidazole (0.5 g/L), and neomycin (1 g/L) were dissolved in autoclaved drinking water using stirring and heating to a maximum of 42 °C. Antibiotic solutions were sterile filtered using a 0.22-µm vacuum filtration system before being administered to mice in black water bottles. Mice were kept on antibiotic drinking water for 14 days, with antibiotic solutions being renewed each fifth day.

Mice were weighed prior to the initiation of antibiotics and then daily during antibiotic administration; any mouse that was found to lose 20% of their pre-antibiotic starting weight was culled in accordance with the humane endpoints of the study.

### Commensal isolate culture and administration to mice

To culture commensal isolates, cryostocks were removed from the − 80 °C freezer and kept on dry ice. Stocks were then scraped using a sterile bacterial loop and the scrapings transferred to a sterile Eppendorf tube on dry ice to minimise exposure to oxygen. Eppendorf tubes were transferred into an anaerobic chamber and scrapings allowed to thaw. Thawed stock cultures were streaked onto YCFA agar plates [55, 56] and incubated at 37 °C until single colonies could be picked — this took between 24 to 96 h depending on the strain. Single colonies were picked and used to inoculate 20-mL fresh YCFA or MRS broth. Broth was incubated at 37 °C until turbid. MRS broth was preferred for culturing *Lactobacillus* species, while YCFA was used for all other species.

For administration to mice, cultures were centrifuged for 10 min at 3900 rpm, the supernatant discarded, and cell pellets resuspended in 3 mL of pre-reduced sterile PBS. Commensal culture was administered 200 µL/mouse via oral gavage. Serial dilutions of inoculating cultures were performed pre- and post-gavage to determine inoculum titres, and 16S rRNA gene PCR and sequencing was performed on colonies to verify the identity of the administered bacterial species.

### *S*. Typhimurium culture and mouse infection

For *S.*Tm culture, cryopreserved stocks were scraped using a sterile bacterial loop and transferred to a sterile Eppendorf tube. *S.*Tm was then plated on LB agar plates and incubated at 37 °C for 24 h under aerobic conditions. A single colony was used to inoculate 10 mL of fresh LB broth which was incubated at 37 °C overnight shaking at 200 rpm. A total of 100 µL of overnight culture was used to inoculate 3 mL of LB broth (1:30 dilution) which was then incubated at 37 °C shaking at 200 rpm for 3 h to reach log-phase growth and a density of 10^9^ colony-forming units (CFU)/mL. The 3-h culture was centrifuged at 3900 rpm for 5 min and the supernatant discarded to remove free endotoxin and secreted metabolites. The pellet was then resuspended in 3 mL of cold sterile PBS.

For infections, the washed *S.*Tm culture was further diluted in cold sterile PBS to obtain 10^7^ CFU/mL (1:100 dilution) for SPF experiments or 10^3^ CFU/mL (1:1,000,000 dilution) for GF experiments. A total of 100 µL of the *S.*Tm inoculum was intragastrically administered to each mouse by oral gavage. All *S.*Tm cultures and inocula were plated via serial dilution to confirm that the expected infectious dose had been given.

Mice were weighed on the day of infection, prior to *S.*Tm oral gavage, and then each morning following infection. Any animal that was found to have lost 20% of their pre-infection starting weight was culled in accordance with the humane endpoints of the study. Physical manifestations of severe disease, i.e. hunching, piloerection, glazed eyes, were also considered as humane endpoints, and any mouse exhibiting these symptoms was culled.

### Screen of MCC isolates in germ-free mice

Selected isolates from the MCC were grown up and administered to GF mice as described above. Faecal pellets were subsequently collected from mice 1 and 2 weeks after colonisation and plated to confirm monocolonisation with the correct isolate and the absence of contamination. Mice were then infected with *S.*Tm 14028 2 weeks after colonisation using the protocol described above. Mice were weighed prior to infection and each day in the morning following infection. Weights at 1-day post-infection (dpi) and the number of days taken to reach 80% of pre-infection weight were used as metrics of infection severity.

### Faecal sample plating and enumeration of faecal titres

Faecal samples were plated to enumerate commensal or pathogen titres, as well as to confirm colonisation and ensure the absence of contamination following monocolonisation of GF mice. Fresh faeces were collected from mice into pre-weighed sterile Eppendorf tubes using aseptic technique. Eppendorf tubes containing faeces were reweighed and the faecal weights recorded. A total of 500 µL of sterile anaerobic PBS was then added and faecal pellets homogenised using a sterile P1000 pipette tip before eight 1:10 serial dilutions were performed for each sample. For each sample, 10 µL of each dilution was plated in triplicate on pre-reduced and pre-warmed agar plates.

For the SPF mouse infection experiments, faecal samples were taken from mice using aseptic technique following cessation of antibiotic treatment, prior to infection, and then each day from 2 dpi. Pre-infection samples were plated on YCFA plates under anaerobic conditions to quantify commensal titres; post-infection samples were plated on *Salmonella Shigella* Agar (SSA; Thermo Fisher Scientific, CM0099) to enable quantification of *Salmonella* titres. Faeces from 1 dpi were not plated.

For GF mouse experiments, faeces were taken 1 week after commensal gavage during cage change and then again at 2 weeks after commensal gavage — the same day as pathogen infection. Samples were plated on YCFA to confirm colonisation and the absence of contamination. Representative colonies for each morphology were picked and processed for 16S rRNA gene sequencing to confirm the identity of the colonising organism.

### 16S rRNA gene PCR and sequencing

Bacterial colonies were picked using a bacterial loop and mixed into a screw cap tube containing DNA-free acid-washed glass beads and 500 µL of sterile PBS. The bead tube was then shaken at speed 6000 for 30 s using a FastPrep Instrument (MPBio). Bead tubes were then centrifuged at 13,200 rpm for 10 min to pellet the cell debris, and the supernatant was taken for the PCR reaction.

GoTaq DNA polymerase kits (Promega) were used for PCR reactions. The 16S rRNA gene was amplified using the broad-range bacterial primers Bact-7F (5′-AGA GTT TGA TYM TGG CTC AG-3′) and Bact-1510R (5′-ACG GYT ACC TTG TTA CGA CTT-3′) which amplify the entire length of the 16S rRNA gene resulting in a PCR product of around 1500 bp. PCR was performed in 50-µL reactions on a T100 thermal cycler (Bio-Rad) for 30 cycles, according to manufacturer’s instructions. Finally, 10 µL of the PCR product was run on a 1% agarose gel (0.8-g agarose + 80-mL TBE) containing SYBR Safe DNA Gel Stain for 15 min at 100 V and 200 A. DNA bands were then visualised using UV light. If the reaction had generated an appropriately sized band, the PCR product was submitted to Eurofins Genomics for PCR product clean up and sequencing.

### Enumeration of Salmonella titres in mouse tissues following infection

GF mice were monocolonised and infected as described above. Mice were culled at 24-h post-infection and whole intestines (jejunum to rectum), mesenteric lymph nodes (mLNs), liver, and spleen removed and kept separately in ice-cold PBS. The gallbladder was separated intact from the liver to avoid contamination with its contents.

The remaining mesenteric and peri-intestinal fat was removed. Samples 0.5–1 cm in length were taken from the jejuno-ileal junction, the terminal ileum, the caecum, and the proximal colon for histology; tissues were placed in tissue cassettes and submerged in methacarn (60% methanol, 30% chloroform, 10% glacial acetic acid). Luminal contents were then collected from the caecum into sterile pre-weighed Eppendorf tubes and stored on ice until weighing and plating. Intestines were next separated into the small intestine, caecum, and colon and processed separately. Each tissue was flayed open longitudinally, luminal contents were gently removed, and tissue was washed sequentially in three petri dishes containing ice-cold PBS.

All tissues were then weighed before being manually homogenised in 0.1% Triton X-100 solution. Livers were added to 6-well plates containing 3-mL 0.1% Triton X-100 solution, while mLNs, spleens, and caeca were added to Eppendorf tubes each containing 500 µL of 0.1% Triton X-100 solution. All tissues were kept on ice throughout this process. Following organ homogenisation, serial dilution of the homogenates was performed and plated on SSA plates to quantify *S.*Tm titres. Caecal contents were weighed before being homogenised in 500 µL of sterile PBS. These samples were briefly spun in a microcentrifuge to settle faecal matter and serial dilutions performed on the supernatants. Serial dilutions were plated on SSA plates to quantify *S.*Tm luminal titres.

### Intestinal epithelial and immune cell isolation

Whole intestines and mLNs were removed from mice and transluminal sections taken from the jejunoileal junction, terminal ileum, caecum, and distal colon for histology. Luminal contents were collected from the terminal ileum, caecum, and distal colon and kept in pre-weighed Eppendorf tubes on ice until plating or further processing. Peyer’s patches were excised from the small intestines prior to processing. The intestines were opened longitudinally, cleared of faeces, and then washed three times in cold PBS. To isolate the intraepithelial immune populations, the intestines were cut into 1-cm pieces, washed in cold PBS, and then incubated in 10 mL of PBS with 1-mM dithiothreitol for 10 min. Tissues were manually disrupted via shaking for 2 min and then strained, collecting the supernatant. Tissues were then incubated in 10 mL of PBS with 30-mM EDTA and 10-mM HEPES at 37 °C in a shaker at 200 rpm for 10 min, before being manually shaken and strained. The supernatants from these two steps were pooled, filtered at 70 µm, and the filtrate fractionated using a discontinuous Percoll gradient (80%/40%). Epithelial cells were isolated from the surface of the Percoll, and the intraepithelial immune cells were isolated from the interface.

To isolate lamina propria immune cells, the intestines were incubated again in 10 mL of PBS with 30-mM EDTA and 10-mM HEPES at 37 °C in a shaker at 200 rpm for 10 min, before being manually shaken and strained. Intestinal tissues were then finely chopped and digested in complete HBSS-2 (HBSS, 25-mM HEPES, 1-mM sodium pyruvate) containing 0.05 mg/mL Collagenase VIII (Sigma) and 50 mg/mL DNase I (Sigma) for 1 h at 37 °C, shaking at 80 rpm. Samples were mechanically disrupted by pipetting through serological pipettes and filtered at 70 µm. The filtrate was fractionated using a discontinuous Percoll gradient (80%/40%). Lamina propria immune cells were isolated from the interface.

Mesenteric lymph nodes were processed using the same protocol. Remaining lamina propria and fat was removed, and tissues were digested in 3-mL complete HBSS-2 containing 0.5 mg/mL collagenase D (Sigma) at 37 °C for 30 min. Tissues were then forced through 70-µm filters to yield single-cell suspensions. Collagenase was quenched with 2-mL complete HBSS-2. Cells were then pelleted, and the supernatant was discarded.

### Flow cytometry

Immune cell populations were characterised by flow cytometry using the gating strategy in Supplementary Fig. 6. Two panels were used to quantify lymphocytes and myeloid cells. Non-viable cells were stained using Live/Dead Fixable Aqua after which surface markers were stained using fluorophore-conjugated primary antibodies (Table 2). For nuclear staining, lymphocytes were permeabilised with Cytofix/Cytoperm (BD) and Foxp3 staining performed in Permeabilization Buffer (eBioscience). Cells were then fixed in 1% paraformaldehyde in preparation for flow cytometric analysis. Sample data were acquired with an Attune NxT flow cytometer coupled with an Attune CytKick Max Autosampler. Data were analysed using FlowJo v10 and R.

**Table 2:** Antibodies for lymphoid and myeloid cell population characterisation by flow cytometry

Cell marker	Fluorophore	Dilution
CD3e	FITC	1:200
B220	FITC	1:200
TCRg	FITC	1:200
CD8b	PE	1:200
SiglecF	PE	1:200
TCRg	PerCP-e710	1:200
CD11b	PerCP-Cy5.5	1:200
CD4	PE-Cy7	1:200
CD11c	PE-Cy7	1:200
B220	APC	1:200
CD64	APC	1:200
CD8a	AF700	1:200
TCRb	APC-Cy7	1:200
MHCII	APC-e780	1:2000
CD45	SB600	1:200
Foxp3	bv421	1:150
Ly6C	e450	1:200
Live/dead	Fixable Aqua	1:1000

### Intestinal histology

For histological analyses, mice were euthanised either prior to infection, on day 14 after commensal monocolonisation, or on day 1 post-infection. Intestinal tissues were excised, and samples around 1 cm in length were taken from the jejuno-ileal junction, terminal ileum, the caecum, and the distal colon. Sections containing luminal contents were preferentially taken. Tissue sections were fixed in methacarn (60% methanol, 30% chloroform, 10% glacial acetic acid) for 18 h at 4 °C and then washed three times in 70% ethanol at room temperature for 10 min each. Samples were stored in 70% ethanol at 4 °C until submission to the histology core facility for paraffin embedding and sectioning (5 µm) and staining with haematoxylin and eosin (H&E) for histology scoring. Additional sections/slides were produced for downstream fluorescence in situ hybridisation (FISH).

### Intestinal pathology scoring

Pathohistological scoring of tissue sections was performed as previously described [33]. Histology slides of colon and caecum cross-sections were scored across five domains by two independent and blinded researchers. The domains scored were (1) degree of submucosal oedema, (2) severity of leukocyte infiltration in lamina propria, (3) epithelial integrity, (4) the presence of cryptitis and crypt abscesses, (5) the number of polymorphonuclear granulocytes (PMN) infiltrating the lamina propria, and (6) the number of goblet cells. Submucosal oedema was assessed using low-power fields (25 × magnification), one for each section. Other domains were assessed across 10 high-power fields (400 × magnification) taken from different regions of each section.

#### Submucosal oedema

- 0 — No pathological changes
- 1 — Detectable oedema (submucosal oedema, < 50% wall thickness)
- 2 — Moderate oedema (submucosal oedema, 50 to 80% wall thickness)
- 3 — Profound oedema (submucosal oedema, ≥ 80% wall thickness)

#### Mononuclear (mixed leukocyte) infiltrate

- 0 — None: normal number of mononuclear cells
- 1 — Low: slightly more than normal infiltration
- 2 — Moderate: many cells present and some evidence of tissue distension
- 3 — High: extreme infiltration, often with tissue architecture disruption

#### Epithelial integrity

- 0 — No pathological changes detectable
- 1 — Epithelial desquamation
- 2 — Erosion of the epithelial surface (gaps of 1 to 10 epithelial cells per lesion)
- 3 — Epithelial ulceration (gaps of > 10 epithelial cells per lesion or granulation tissue below the epithelium)

#### Cryptitis and crypt abscesses

- 0 — None: no neutrophils between epithelial cells or in crypts
- 1 — Low: some evidence of neutrophil infiltration of the crypt epithelium
- 2 — Moderate: many neutrophils between crypt epithelial cells, some in the crypts
- 3 — High: abundant crypt abscesses (neutrophils in the crypts)

#### PMN infiltration into the lamina propria

- 0 — < 5 PMN/high-power field
- 1 — 5 to 20 PMN/high-power field
- 2 — 21 to 60 PMN/high-power field
- 3 — 61 to 100 PMN/high-power field
- 4 — > 100 PMN/high-power field

N.B. Where transmigration of PMN into the intestinal lumen was observed, sections were given a score of at least 3.

#### Goblet cells

- 0 — > 28 Goblet cells/high power
- 1 — 11 to 28 Goblet cells/high-power field
- 2 — 1 to 10 Goblet cells/high-power field
- 3 — < 1 Goblet cell/high-power field

For each sample, the two independent scores across all sections were averaged for each domain, and the combined pathological score for each sample was determined as the sum of these averaged scores.

### Fluorescence in situ hybridisation

FISH was performed as previously described [57, 58] using a universal bacterial FISH primer, Eub338 (5′-GCT GCC TCC CGT AGG AGT-3′), conjugated to FITC at both termini.

Fresh hybridisation buffer (0.9-M NaCl, 20-mM Tris–HCl [pH 7.2], 0.1% SDS) was warmed to 50 °C and used to dilute the bacterial DNA probe 1:300. A total of 50 µL/slide of staining solution was added directly onto deparaffinised tissue slides and then gently covered with a piece of parafilm cut to the size of a coverslip. Slides were incubated in a humidified box shielded from light for 3 h at 50 °C. Tissues were then washed twice in warm wash buffer (0.9-M NaCl, 20-mM Tris–HCl [pH 7.2]) for 10 min each. Next, Hoechst stain was diluted 1:3000 in wash buffer and 100 µL added directly to slides. Slides were incubated for 5 min at room temperature while shielded from light before being washed once more for 10 min in wash buffer. Slides were then thoroughly dried, one drop of ProLong Diamond Antifade Mountant directly applied to each slide, and a cover glass carefully positioned. Slides were kept at 4 °C overnight prior to imaging.

### Immunofluorescence of *S*. Typhimurium

Immunofluorescence was performed on post *S.*Tm infection samples to visualise the interaction of *S.*Tm with the intestinal epithelium. *S.*Tm was stained using rabbit antiserum against the O4 antigen (TR1302, Sifin), and phalloidin-AF488 (ab176753, Abcam) and DAPI (D1306, Thermo Fisher Scientific) were used to visualise the actin cytoskeleton and host nuclei, respectively.

Slides were deparaffinised and kept in PBS for 30 min. For heat-induced epitope retrieval, slides were incubated in sodium citrate buffer (10-mM Sodium citrate, 0.05% Tween 20, pH 6.0) at 95 °C for 20 min. Slides were subsequently allowed to cool to room temperature for 30 min after which they were washed in Milli-Q Water for 5 min and then PBS for 10 min. Next, slides were incubated in blocking buffer (2% BSA, 0.5% Triton in PBS) for 20 min at room temperature. Rabbit anti-O4 primary antibody was diluted 1:10 in blocking buffer, and 50 μL was added directly to slides and left to stain overnight at 4 °C. Slides were then washed three times in PBS for 5 min each. Anti-rabbit-AF568 (A-11011, Invitrogen)) was diluted 1:1000 in blocking buffer, and phalloidin-AF488 conjugate was added to yield a final concentration of 1 × . A total of 50 μL of secondary antibody and phalloidin staining mixture was added directly to slides and incubated at room temperature for 2 h. Slides were then washed three times in PBS for 5 min each. Finally, 50 μL of 300-nM DAPI solution was added to each slide and incubated for 5 min. Slides were washed three times in PBS for 5 min each and then dried thoroughly. One drop of ProLong Diamond Antifade mounting medium was directly applied to each sample and a cover glass carefully positioned to prevent bubble formation. Slides were kept at 4 °C overnight prior to imaging.

### Confocal microscopy and image analysis

FISH and immunofluorescence slides were visualised using a Leica TCS SP8 lightning confocal laser scanning microscope (Leica Microsystems). For figure production, images were processed using Fiji-2. Auto-adjustment of brightness and contrast was used to optimise colour handling in captured images.

### Scoring epithelial proximity by commensal microbes

Commensal microbe interactions with the intestinal epithelium were qualitatively ascertained using a previously published epithelial proximity score [59]. Ten high-power fields were taken of each section and scored by an independent, blinded researcher according to the below rubric:

- 0 — Bacteria are completely excluded from the proximity of the epithelium.
- 1 — Some bacteria can be seen in close proximity to the epithelium, but there is no contact.
- 2 — Many bacteria can be seen in close proximity to the epithelium with sporadic contact; however, there is still clear evidence of a separation between the bacteria and the epithelium.
- 3 — Many bacteria can be seen in close proximity to the epithelium with many contact points, and there is less evidence of a clear separation between bacteria and epithelium.
- 4 — Significant contact of bacteria with the epithelium, with limited evidence of separation between bacteria and the epithelium
- 5 — Complete contact of the bacteria with the epithelium

### In vitro colonisation resistance assays

*S.*Tm growth was tracked using CFU quantification in commensal isolate stationary phase broth culture and sterile-filtered spent media, while OD was used as a proxy for growth in the spent media glucose rescue assays.

For all protocols, commensal isolates were grown on agar as described above and single colonies inoculated into pre-reduced LB broth which was then incubated at 37 °C under anaerobic conditions for 36 h to reach stationary phase. Growth curves were generated for all isolates using CFU/mL to confirm that isolates grew in LB and determine the time required to reach stationary phase. For the spent media assays, stationary phase media was centrifuged at 3900 rpm for 10 min and the supernatant sterile filtered using 22-µm filters into sterile 50-mL tubes. Stationary phase media and spent media were each split into three 10-mL aliquots. For the glucose rescue assays, spent media was split into 2-mL aliquots and sterile 10% glucose solution added to aliquots to make up final glucose concentrations of 0%, 0.02%, 0.05%, 0.1%, and 0.2%. Following this, 200 µL of each concentration was added in triplicate to a flat-bottom 96-well plate.

For *S.*Tm inoculation, a fresh *S.*Tm preparation of 10^8^ CFU/mL was added 1:100 to each aliquot or well. For stationary phase and spent media experiments, inoculated media was then incubated without shaking under anaerobic conditions at 37 °C for 24 h. At 4-, 8-, and 24-h timepoints, tubes were gently swirled, 100 µL of each culture was taken, and serial dilutions were performed. Serial dilutions were plated on SSA (stationary) or LB (spent) plates to quantify *S.*Tm CFU titres. For glucose rescue assays, *S.*Tm was directly inoculated into wells of the 96-well plate. The OD of each well was then measured after 24 h of stationary incubation at 37 °C under anaerobic conditions.

### Ex vivo faecal spiking assay

Fresh caecal contents were collected from GF and gnotobiotic mice monocolonised with either *E. clostridioformis*, *E. coli*, or *A. faecis* into pre-weighed Eppendorf tubes. Tubes containing luminal contents were then reweighed and rapidly transferred to an anaerobic cabinet. A total of 500 µL of pre-reduced sterile PBS was added to each sample. Samples were homogenised, and 200 µL of the caecal slurry was taken to perform the ex vivo faecal spiking assay.

For faecal spiking assays, a preparation of 10^6^ CFU/mL *S.*Tm was prepared under aerobic conditions as described above. A total of 20 µL was added to homogenised samples such that the final *S.*Tm titre was 10^5^ CFU/mL. Spiked faecal samples were then incubated under anaerobic conditions at 37 °C for 24 h. To quantify *S.*Tm titres throughout the experiment, serial dilutions were performed at 4-, 8-, and 24-h timepoints using 10 µL of each sample. Serial dilutions were plated on preheated SSA plates and then incubated under aerobic conditions at 37 °C for 24 h until colonies were quantified.

### *S*. Typhimurium virulence gene expression assay

Caecal contents were spiked with *S.*Tm, as described above, and then incubated under anaerobic conditions at 37 °C for 18 h. RNA was extracted from caecal contents using a previously published protocol [60]. Briefly, 1 mL of TRIzol reagent per 100-µL caecal contents was added to samples. Samples were mixed by pipetting and vortexing before being allowed to sit for 10 min. Samples were then centrifuged at 4 °C for 10 min at 12,000 × g to pellet insoluble material. The supernatant was transferred to new RNase-free Eppendorf tubes and immediately processed for RNA extraction from TRIzol according to the manufacturer’s instructions.

### MODE-K invasion assay

A modified in vitro cell invasion assay based on Gantois et al. 2006 [23] was used to quantify intracellular *S.*Tm following pretreatment of MODE-K cells with commensal spent media. 10^6^ MODE-K cells were cultured for 3 days in 24-well plates containing sterile-filtered growth medium (DMEM with 4.5 g/L D-glucose (Gibco), 10-mM HEPES, 100 U/mL penicillin & 100 µg/mL streptomycin, 0.4 mg/mL L-glutamine, 1 × non-essential amino acids (Gibco), 10% heat-inactivated foetal bovine serum, 50-µM β-mercaptoethanol), supplemented with 15% LB media or sterile spent media from *E. clostridioformis* or *E. coli*. Cells were incubated statically at 37 °C. *S.*Tm was grown overnight and subcultured for 3 h as per the infection protocol described above. *S.*Tm pellets were resuspended in DMEM and added to MODE-K cells at a multiplicity of infection of 10. Plates were centrifuged at 1500 rpm for 5 min at 21° before being incubated statically for 30 min at 37 °C and 5% CO2. Extracellular bacteria were then killed by replacing media with DMEM containing 30 μg/ml gentamicin for 30 min. The gentamicin concentration was subsequently reduced to 5 μg/ml for 4 h. After 4 h, cell media was removed, and cells were washed three times in D-PBS. The infected cells were then lysed in 0.1% sodium dodecyl sulphate and intracellular populations determined by viable counts.

### RNA extraction from intestinal epithelial cells

IECs were isolated during the first Percoll gradient step during the immune cell isolation protocol. The epithelial cell layer was pipetted from the surface of the Percoll gradient using a P1000 pipette and transferred to a 15-mL Falcon tube containing 8 mL of HBSS-2. IECs were centrifuged at 4 °C for 5 min at 2000 rpm and the supernatant discarded. IECs were then resuspended in 1 mL of HBSS-2 and transferred to a PCR-grade 1.5-mL Eppendorf tube. Tubes were centrifuged at 4 °C for 1 min at 900 × g and supernatant discarded.

The cell pellet was resuspended in 1 mL of TRIzol (Invitrogen), pipetting up and down until it was completely homogenised. The tubes were vortexed briefly before being centrifuged at 4 °C for 10 min at 12,000 × g to pellet fat and insoluble material. The top soluble fraction was then transferred to a new Eppendorf tube and immediately frozen at − 80 °C until RNA extraction. RNA was extracted from TRIzol according to the manufacturer’s instructions.

RNA concentration and purity were calculated using a NanoDrop. For each sample, RNA concentrations were diluted to 200 ng/µL in RNase-free water. An A260:A280 ratio over 1.8 was considered high purity. If RNA was low purity, then a clean-up protocol was implemented. For this, RNA sample volumes were made up to 500 µL with RNase-free water, and 50 µL of 3-M sodium acetate (pH 5.5) was added with 10-µg glycogen, depending on whether it was a low-yield sample. A total of 500 µL of isopropanol was then added, the tube mixed well, and then incubated for 20 min. RNA was pelleted by spinning at 12,000 × g at 4 °C and supernatant removed. The pellet was then washed twice with 300–500 µL ice-cold ethanol. Finally, the RNA pellet was left to air dry for 15 min before resuspension in RNase-free water.

### RNA quantification by reverse transcription-quantitative polymerase chain reaction

Sequences for each gene of interest were accessed via UniProt and then analysed as a PCR template using the NCBI Primer-BLAST web interface tool [61]. Primers were designed to produce a PCR product with an amplicon size of 70–200 bp and were checked for specificity against *Mus musculus* RefSeq mRNA database (for IEC genes) or against the RefSeq mRNA database for the domain Bacteria (for *S.*Tm virulence genes). Results for potential primers were then manually screened, and the most optimal primer pairs were chosen that minimised predicted self-complementarity. For *S.*Tm virulence genes, all primers were screened against RNA from *E. coli* and *E. clostridioformis* to ensure specificity. The sequences for primers used in this study are provided in Table 3 and Table 4.

**Table 3:** qPCR primers for IEC gene expression. All primer sequences are provided for the 5′–3′ direction

Gene target	Forward primer	Reverse primer
*Aldh1a1*	AGAGGTTTGCACCGTCAAGA	GACTGCGTCCAGAGTGACTG
*Fut2*	ACCTCCAGCAACGAATAGTGA	TGAGGACATTTGAACCGCCT
*Hprt1*	GTTGGGCTTACCTCACTGCT	TCATCGCTAATCACGACGCT
*Il6*	TCCTACCCCAATTTCCAATGCT	TGGTCTTGGTCCTTAGCCAC
*Ido1*	ATGTGGGCTTTGCTCTACCA	AGCTGCCCGTTCTCAATCAG
*Muc2*	GGCCAGGAGTTTACCAACGA	CAGGGCAAGGCAGGTCTTTA
*Nlrc4*	AGGTCACAGAAGAAGACCTGAAT	ACCCAGGGGGTAGAAGTTCA
*Ocln*	TTGAAAGTCCACCTCCTTACAGA	AGAGTACGCTGGCTGAGAGA
*Reg3g*	CAGACAAGATGCTTCCCCGT	GCAACTTCACCTTGCACCTG
*Retnlb*	CAAGGAAGCTCTCAGTCGTCA	CAACCATCCCAGCAGGACAG
*Rpl13*	AGAAGGGAGACAGTTCTGCTG	TTGCTCGGATGCCAAAGAGT
*Saa1*	TGAAGGAAGCTAACTGGAAAAACTC	CCTCTGCCGAAGAATTCCTGA
*Tjp1*	GCCGCTAAGAGCACAGCAA	AAATCCAAACCCAGGAGCCC
*Tnf*	CCTCACACTCACAAACCACCA	ACAAGGTACAACCCATCGGC

**Table 4:** qPCR primers for *S.*Tm virulence gene expression. All primer sequences are provided for the 5′–3′ direction

Gene target	Forward primer	Reverse primer
*gyrB*	CGTCACTGGCGATACCGATAA	GTTGGTGAAGGTTTCGTGGC
*hilA*	GGGCAGATGATACCCGATGG	AAGAGAGAAGCGGGTTGGTG
*hilD*	TGCTTTCGGAGCGGTAAACT	CCAACATCCCAGGTTCGTCA
*invF*	TCGATGGCGCAGGATTAGTG	TATTGAAGGCCGGAGAAGGC
*sirC*	CCCGGTGGGTTTGATTTCCT	GCGGGTGAGATCGCTGATAA
*rtsA*	ACTCATCAAGCTCACCACGG	ATTGGCCCTGATGCCGTAAT
*sipC*	CAGCTTCGCAATCCGTTAGC	GACTCAACCCCAGGTCACTG
*sopB*	GAGGCGGTAAGCCTGAAACT	TCGTTCCCTCTTTGCCGAAA
*prgH*	GGAGGACGCTATGTGCAGTT	CTTCCGCCCCGTACTGAAAT

To perform RTqPCR, cDNA was first synthesised from the extracted RNA using the Bio-Rad iScript kit and a T100 thermal cycler (Bio-Rad) as per the manufacturer’s instructions. Resulting cDNA was diluted to 5 ng/µL in nuclease-free water prior to qPCR. qPCR reactions were performed in 384-well plates using the iTaq Universal SYBR Green Supermix (Bio-Rad) as per manufacturer’s instructions on a QuantStudio 7 Flex qPCR machine (‘fast’ mode, 40 cycles). Data were exported and analysed in R. All runs were quality controlled using melt curve profiles. Low-quality runs, with unexpected melt curve patterns including bimodal melt curves and different peak melt temperatures from reference, were excluded from analyses.

The mean Ct value for two housekeeping genes, *rpl13* and *hprt1*, was calculated per sample, and the relative expression of test genes was calculated using the formula as follows:

$$10000\times 2{ }^{C{t}_{hkg} - C{t}_{test}}$$

where *C*t_hkg_ is the mean Ct value of the housekeeping genes and *Ct*_test_ is the Ct value of the target gene of interest. Relative expression levels of genes were then compared between treatment groups using *t*-tests.

### Bulk RNA sequencing and analysis

RNA from caecal epithelial cells from six mice for each GF, *E. coli*, *E. clostridioformis*, and SPF treatment groups were selected from across three experiments (two per experiment). Samples were selected on the basis of having high-quality RNA extraction and being associated with a representative weight loss phenotype. A minimum of 100-ng total RNA per sample was sequenced using the Illumina HiSeq 4000 System, with 150-bp paired-end sequencing and a total yield of 350 million reads per lane. Sequencing was performed at Wellcome Sanger Institute, Hinxton, UK.

Bulk RNA-seq data was aligned to the mm10 transcriptome using Salmon [62]. Differential gene expression testing was performed using DESeq2 [63]. Metadata for RNAseq samples are provided in Supplementary Table 2. Volcano plots were generated in R. Meaningfully differentially expressed genes were defined as having an *FDR* ≤ 0.05 and absolute log_2_ fold change ≥ 1.5. GO term enrichment analyses were performed using PANTHER (v18.0) [64, 65], and GO terms were ranked according to FDR adjusted *p*-values.

## Supplementary Information

Supplementary Material 1: Supplementary figures: Supplementary Fig. 1. The gut microbiota is essential for resistance against *S.* Typhimurium infection but can confound mouse studies. (A) Weight loss (left) and survival (right) data for C57BL/6NTac mice infected with *S.*Tm following antibiotic administration in drinking water (d.w.) or by intragastric gavage (i.g.). Data shown are pooled from five experiments (*n* = 3–18). (B) Faecal microbiota titres at two days post cessation of antibiotics correlate with duration of survival following *S.*Tm infection. Points represent individual mice. This subfigure combines data from both d.w. and i.g. protocols. Linear correlation was assessed using the Pearson product-moment correlation coefficient. Data are pooled from three independent experiments. (C) Titres of faecal commensal bacteria in the days following antibiotic treatment in the drinking water. Each bar represents the mean faecal titres from a different cage of mice from one experiment. Error bars indicate SEM. Supplementary Fig. 2. *S.*Tm virulence gene expression is unaffected by *E. clostridioformis*. (A) Relative expression of *S.*Tm virulence genes following 18 h culture in caecal contents from GF, *E. clostridioformis*-, or *E. coli*-monocolonised mice. Data shown are pooled from three experiments (*n* = 10–11). Significance was assessed using student’s t-tests. Error bars indicate SEM. (B) Relative expression of *S.*Tm virulence genes following 18 h culture of *E. coli* or *S.*Tm in LB broth (*n* = 2). Supplementary Fig. 3. *E. clostridioformis* is associated with less pathogen–epithelium contact in vivo. (A) Quantification of *S.*Tm contact with the caecal epithelium (CFU/mm epithelium) at 1 dpi (*n* = 8–12). (B) Representative micrographs of caecal epithelium 1dpi stained for S.Tm (anti-O4 antigen, red), and host nuclei (DAPI, blue) and cytoskeleton (phalloidin, blue). (C) Intracellular *S.*Tm titres using an in vitro MODE-K invasion assay (*n* = 2). MODE-K cells were pretreated for 72 h with LB or sterile bacterial spent media from *E. clostridioformis* or *E. coli* prior to infection. Wilcoxon tests were used to assess statistical significance in (A) and (C). Supplementary Fig. 4. Epithelial architecture is maintained after *E. clostridioformis* monocolonisation. (A) Relative expression by qPCR of host defence genes in caecal epithelial cells isolated from GF, SPF, or *E. clostridioformis*-, *E. coli*-, or *A. faecis*-monocolonised mice. Data shown are pooled from six experiments (*n* = 4–14). Significance was assessed using student’s t-tests with Benjamini–Hochberg correction. Error bars indicate SEM. (B) Quantification of caecal epithelium thickness from GF, SPF, and *E. clostridioformis*- and *E. coli*-moncolonised mice. Data are pooled from 5 experiments (*n* = 4–6). Significance was assessed using Wilcoxon tests. (C) Representative micrographs showing the caecal epithelium architecture. Mice were monocolonised with *E. clostridioformis* for two weeks prior to sampling. Host cells were stained with Hoescht and micrographs taken using confocal microscopy. Supplementary Fig. 5. Flow cytometric data for colon and mesenteric lymph node compartments. Flow cytometric quantification data for additional lymphocyte populations from the colon intraepithelial compartment and lamina propria as well as mesenteric lymph nodes. Each data point represents a single mouse. Pooled data are shown from three experiments (*n* = 10–12). Wilcoxon tests with Benjamini–Hochberg correction. Bars and error bars represent median ± median absolute deviation, respectively. Supplementary Fig. 6. Gating strategy for flow cytometric analysis of intestinal lymphocytes.

Supplementary Material 2: Supplementary tables: Supplementary Table 1: GO term enrichment from caecal epithelial cell RNAseq analyses. Supplementary Table 2: Metadata for RNAseq samples.

## Data Availability

RNA sequencing data are deposited in the European Nucleotide Archive under project accession PRJEB75752 (ebi.ac.uk/ena/browser/view/PRJEB75752). The data and code used to generate analyses and figures are available on the project’s GitHub page (github.com/Pedicord-Lab/Microbiota-in-Salmonella-infection). All other data analysed during this study are included within the manuscript and its supplementary information files.

## Abbreviations

GF: Germ-free
SPF: Specific pathogen-free
*S.*Tm: *Salmonella enterica* serovar Typhimurium
DNA: Deoxyribonucleic acid
RNA: Ribonucleic acid
CFU: Colony-forming unit
SSA: *Salmonella Shigella* Agar
LB: Luria-Bertani
dpi: Day(s) post-infection
RTqPCR: Reverse transcription-quantitative polymerase chain reaction
PCR: Polymerase chain reaction
FISH: Fluorescence in situ hybridisation
IEC: Intestinal epithelial cell
IEL: Intraepithelial lymphocyte
LPL: Lamina propria lymphocyte
OD: Optical density
NTS: Non-typhoidal salmonellosis
FDR: False discovery rate
bp: Base pairs
PMN: Polymorphonuclear granulocytes
MCC: Mouse Culture Collection
SEM: Standard error of the mean
