# Supplementary material for: Enterocloster clostridioformis protects against Salmonella pathogenesis and modulates epithelial and mucosal immune function
Source: Microbiome. 2025 Feb 28;13:61. doi: 10.1186/s40168-025-02050-9 (PMC11869688; doi:10.1186/s40168-025-02050-9)
Supplement: Supplementary file 2 — Supplementary Material 1: Supplementary figures: Supplementary Fig. 1. The gut microbiota is essential for resistance against S. Typhimurium infection but can confound mouse studies. (A) Weight loss (left) and survival (right) data for C57BL/6NTac mice infected with S.Tm following antibiotic administration in drinking water (d.w.) or by intragastric gavage (i.g.). Data shown are pooled from five experiments (n = 3–18). (B) Faecal microbiota titres at two days post cessation of antibiotics correlate with duration of survival following S.Tm infection. Points represent individual mice. This subfigure combines data from both d.w. and i.g. protocols. Linear correlation was assessed using the Pearson product-moment correlation coefficient. Data are pooled from three independent experiments. (C) Titres of faecal commensal bacteria in the days following antibiotic treatment in the drinking water. Each bar represents the mean faecal titres from a different cage of mice from one experiment. Error bars indicate SEM. Supplementary Fig. 2. S.Tm virulence gene expression is unaffected by E. clostridioformis. (A) Relative expression of S.Tm virulence genes following 18 h culture in caecal contents from GF, E. clostridioformis-, or E. coli-monocolonised mice. Data shown are pooled from three experiments (n = 10–11). Significance was assessed using student’s t-tests. Error bars indicate SEM. (B) Relative expression of S.Tm virulence genes following 18 h culture of E. coli or S.Tm in LB broth (n = 2). Supplementary Fig. 3. E. clostridioformis is associated with less pathogen–epithelium contact in vivo. (A) Quantification of S.Tm contact with the caecal epithelium (CFU/mm epithelium) at 1 dpi (n = 8–12). (B) Representative micrographs of caecal epithelium 1dpi stained for S.Tm (anti-O4 antigen, red), and host nuclei (DAPI, blue) and cytoskeleton (phalloidin, blue). (C) Intracellular S.Tm titres using an in vitro MODE-K invasion assay (n = 2). MODE-K cells were pretreated for 72 h with [file 40168_2025_2050_MOESM1_ESM.zip › Stm Fig S1_ESM.pdf]

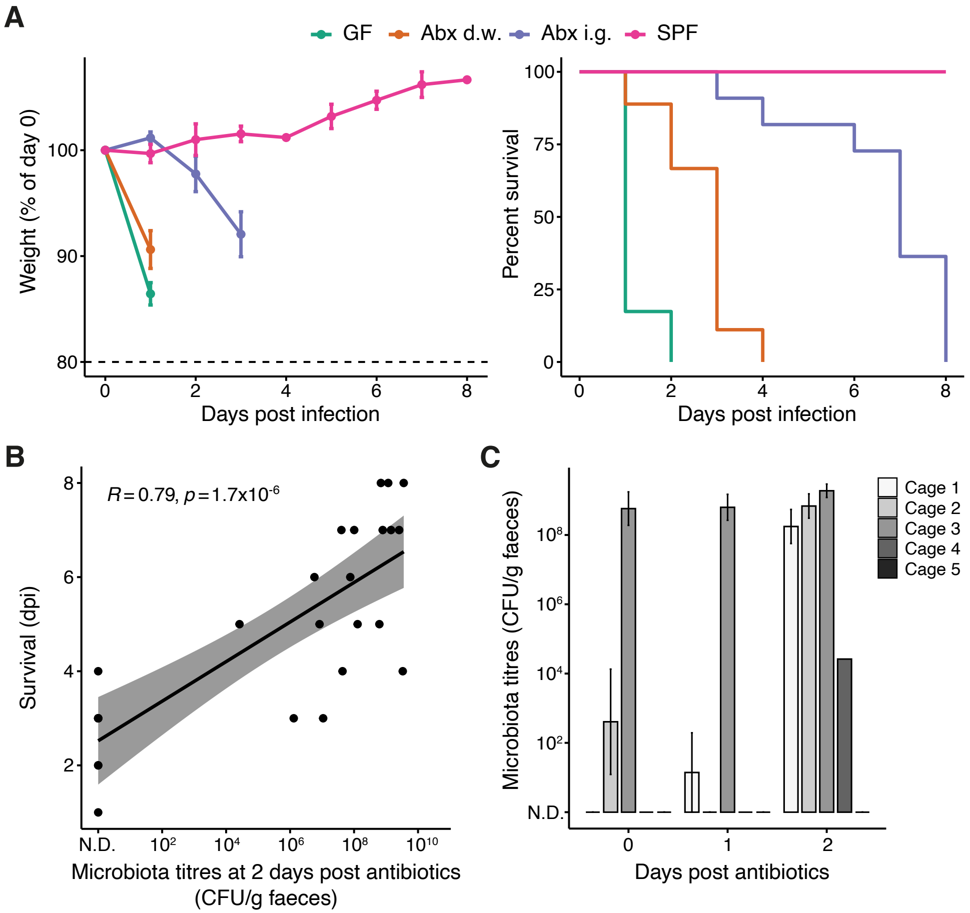

Supplementary Figure 1. The gut microbiota is essential for resistance against *Salmonella* Typhimurium infection but can confound mouse studies. (A) Weight loss (left) and survival (right) data for C57BL/6NTac mice infected with *S.*Tm following antibiotic administration in drinking water (d.w.) or by intragastric gavage (i.g.). Data shown are pooled from five experiments (n=3–18). (B) Faecal microbiota titres at two days post cessation of antibiotics correlate with duration of survival following S.Tm infection. Points represent individual mice. This subfigure combines data from both d.w. and i.g. protocols. Linear correlation was assessed using the Pearson product-moment correlation coefficient. Data are pooled from three independent experiments. (C) Titres of faecal commensal bacteria in the days following antibiotic treatment in the drinking water. Each bar represents the mean faecal titres from a different cage of mice from one experiment. Error bars indicate SEM.
